# Fusaripyridines A and B; Highly Oxygenated Antimicrobial Alkaloid Dimers Featuring an Unprecedented 1,4-Bis(2-hydroxy-1,2-dihydropyridin-2-yl)butane-2,3-dione Core from the Marine Fungus *Fusarium* sp. LY019

**DOI:** 10.3390/md19090505

**Published:** 2021-09-06

**Authors:** Lamiaa A. Shaala, Torki Alzughaibi, Grégory Genta-Jouve, Diaa T. A. Youssef

**Affiliations:** 1 Natural Products Unit, King Fahd Medical Research Center, King Abdulaziz University, Jeddah 21589, Saudi Arabia; 2 Department of Medical Laboratory Sciences, Faculty of Applied Medical Sciences, King Abdulaziz University, Jeddah 21589, Saudi Arabia; taalzughaibi@kau.edu.sa; 3 Suez Canal University Hospital, Suez Canal University, Ismailia 41522, Egypt; 4 King Fahd Medical Research Center, King Abdulaziz University, Jeddah 21589, Saudi Arabia; 5 UMR 8038 CiTCoM, Faculté de Pharmacie de Paris, Université Paris Descartes, Avenue de l’observatoire, 75006 Paris, France; gregory.genta-jouve@parisdescartes.fr; 6 Molecules of Communication and Adaptation of Microorganisms (UMR 7245), National Museum of Natural History, CNRS, 75231 Paris, France; 7 Department of Natural Products, Faculty of Pharmacy, King Abdulaziz University, Jeddah 21589, Saudi Arabia

**Keywords:** Red Sea Verongiid sponge, *Suberea mollis*, marine-derived fungus, *Fusarium* sp. LY019, marine alkaloid dimers, fusaripyridines A and B, antimicrobial activity, *C. albicans*, cancer cell growth inhibition, HeLa cells

## Abstract

The fungal strain, *Fusarium* sp. LY019, was obtained from the Red Sea sponge *Suberea mollis*. Bioassay-directed partition of the antimicrobial fraction of the extract of the culture of the fungus provided two dimeric alkaloids, fusaripyridines A and B (**1** and **2**). The compounds possess a previously unreported moiety, 1,4-bis(2-hydroxy-1,2-dihydropyridin-2-yl)butane-2,3-dione. Further, the compounds display a highly oxygenated substitution pattern on the dihydropyridine moieties, representing an additional feature of the fusaripyridines. Fusaripyridines A and B are the first examples of natural products possessing 1,4-bis(2-hydroxy-1,2-dihydropyridin-2-yl)butane-2,3-dione backbone. Careful analyses of the one- and two-dimensional NMR and HRESIMS spectra of the compounds secured their structural mapping, while their absolute stereochemistry was established by analyses of their ECD spectra. The production of such dimeric alkaloids with an unprecedented moiety in the culture of *Fusarium* sp. LY019 supports further understanding of the biosynthetic competences of the cultured marine-derived fungi. Fusaripyridines A and B selectively inhibited the growth of *Candida albicans* with MIC values down to 8.0 µM, while they are moderately active against *S. aureus*, *E. coli* and HeLa cells.

## 1. Introduction

Investigation of fungi of marine origin has been mostly ignored for several years as a result of their low occurrence and the doubt about their true existence. Recently, this situation has been changed due to the appreciation that marine-derived fungi embody a relatively diverse class and represent an enormous source of bioactive secondary metabolites. It was found that altering the culture conditions of the fungi, such as addition of sodium chloride for example, stimulates the production of new secondary metabolites not reported under regular culture conditions [1,2,3]. Several cultivation-dependent investigations showed that marine algae and sponges, for example, represent a massive source for fungi [2,4,5,6,7]. Moreover, an unpredicted high fungal variety was found in deep-sea hydrothermal ecosystems using molecular approaches [8]. Recently, a growing number of new fungal secondary metabolites with promising pharmaceutical bioactivities has been discovered, representing potential candidates for drug development. The biotechnological potential of the endophytic fungi has been demonstrated in different applications of the life science as antiviral agents, antifungal agents, antibacterial agents, anticancer drugs and in the biological control of different contaminants and plague [9,10]. Such biological properties are attributed to the unique and diverse fungal secondary metabolites with biotechnological applications and pharmaceutical importance [10,11].

Members of the genus *Fusarium* are widely spread in soils and plants worldwide and represent a central source of plant pathogens in agriculture [12,13]. They are also considered as a rich source of bioactive compounds. Representative examples of *Fusarium*-derived natural products include fusarins A, C, D, and F [14] and lucilactaene [15]. Lucilactaene, a metabolite of the *Fusarium* species, has received widespread attention in terms of both biosynthesis investigation and total chemical synthesis [16,17]. Lucilactaene is considered as inhibitor of cell cycle in p53-transfected cancer cells [15]. 

As a part of ongoing work to identify bioactive compounds of marine microbial origin [18,19,20,21,22,23,24], the antifungal fraction of the extract of the culture of *Fusarium* sp. LY019 was studied. Two novel alkaloid dimers, fusaripyridines A and B (**1** and **2**) with previously unreported highly oxygenated 1,4-bis(2-hydroxy-1,2-dihydropyridin-2-yl)butane-2,3-dione moiety, were isolated and their structures were characterized. Herein, we report about the isolation, structural determinations and the bioactivities of fusaripyridines A and B.

## 2. Results and Discussion

### 2.1. Purification of Fusaripyridines A and B (**1** and **2**)

**Figure 1:**
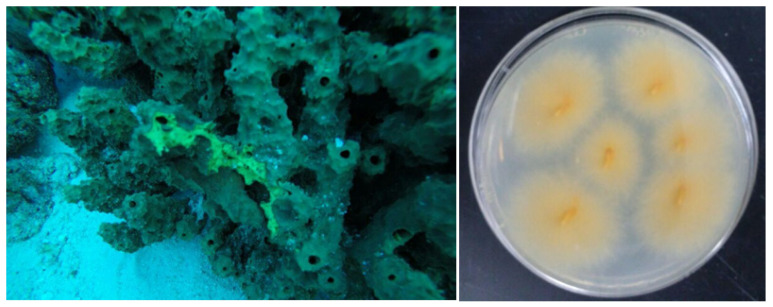
Photograph of the sponge *Suberea mollis* (**left**) and *Fusarium* sp. LY019 (**right**).

**Figure 2:**
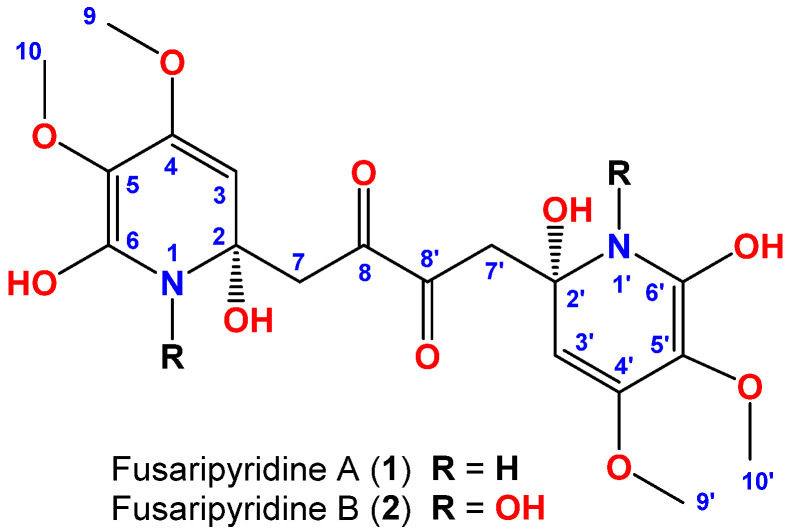
Chemical structures of **1** and **2**.

The marine-derived fungus *Fusarium* sp. LY019 (Figure 1) was obtained from the inner tissue of the sponge *Suberea mollis* (Figure 1). Chromatographic purification of the antimicrobial fraction of the extract of the fermentation broth afforded two alkaloids, fusaripyridines A and B (**1** and **2**) (Figure 2). 

### 2.2. Structure of Fusaripyridine A (**1**)

Fusaripyridine A (**1**) (Figure 2), an optically active compound, with molecular formula C_18_H_24_N_2_O_10_ is supported by HRESIMS (Figure S1), signifying eight degrees of unsaturation. Analysis of its ^1^H (Figure S2), ^13^C (Figure S3) NMR spectra and COSY (Figure S4), HSQC (Figure S5), HMBC (Figure S6) and NOESY (Figure S7) experiments proofed its planar structure. Its ^13^C NMR spectrum revealed nine signals only (Table 1), suggesting a symmetric dimer. The ^13^C NMR data and the HSQC experiment allowed the assignment of the carbons into one methylene, an olefinic methine, two methoxyls (δ_C_ 59.6 and 52.4), an oxygenated quaternary carbon (δ_C_ 78.2), three oxygenated aromatic carbons (132.9–166.6 ppm) and one ketone (δ_C_ 202.1). Furthermore, its ^1^H NMR spectrum exhibited resonances for an olefinic methine (δ_H_ 4.98, s), one methylene (δ_H_ 2.88 and 2.19, each doublet with a geminal coupling of 18.5 Hz) and two methoxyls (δ_H_ 4.01 and 3.82, each s) (Table 1). These signals are associated in the HSQC spectrum to the ^13^C NMR signals at δ_C_ 99.9 (CH), 44.8 (CH_2_), 59.6 (CH_3_) and 52.4 (CH_3_), respectively. 

**Figure 3:**
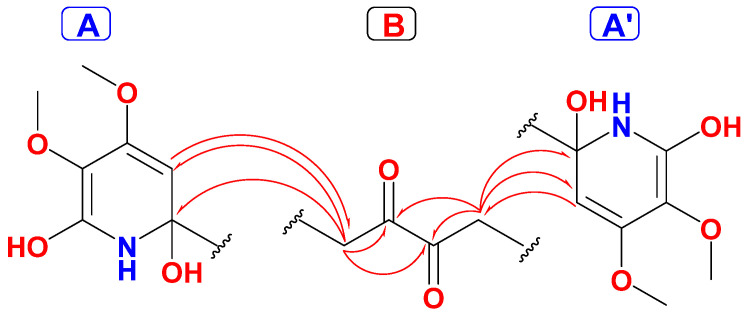
Structural subunits and HMBC connecting subunits of **1**.

**Figure 4:**
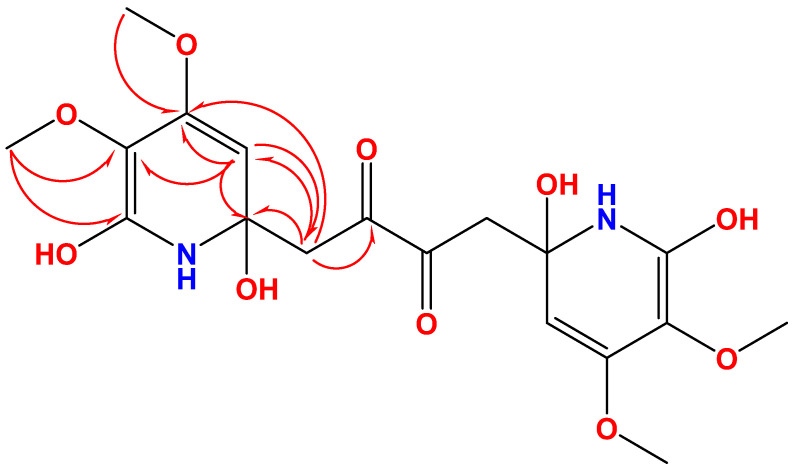
HMBC correlations of **1**.

The COSY, HSQC along with the HMBC experiments allowed the assignment of three fragments within **1** including two identical fragments, namely 2,4,5,6-tetrasubstituted-1,2-dihydropyridine moieties (subunits A and A’) connected symmetrically via C-2, and C-2’ with a 1,4-disubstituted butane-2,3-dione moiety (subunit B) (Figure 3). The methylene signals at δ_H/C_ 2.88 (d, *J* = 18.5 Hz) and 2.19 (d, *J* = 18.5 Hz)/44.8 (CH_2_, H_2_-7/7’ and C-7,7’) together with the ketone functionality at δ_C_ 202.1 (C=O, C-8/8’) constitute subunit B. The HMBC cross-peak from H_2_-7/ H_2_-7’ to the ketone moieties (δ_C_ 202.1, C-8/8’) supported this assignment (Table 1 and Figure 3 and Figure 4). The subunit A and A’ are assigned as 2,6-dihydroxy-4,5-dimethoxy-1,2-dihydropyridinyl moiety as supported by the signals at δ_H/C_ 4.98/99.9 (CH, C-3/3’), 158.5 (qC, C-4/4’), 132.2 (qC, C-5/5’), 166.6 (qC, C-6/6’), 4.01/59.6 (2 × OCH_3_, H_3_-9/9’ and C-9/9’) and 3.82/52.4 (2 × OCH_3_, H_3_-10/10’ and C-10/10’). 

The HMBC correlations from H_2_-7/7’ to C-2/2’ and C-3/3’ and from H-3/3’ to C-7/7’ connect subunits A and A’ with subunit B and confirm the placement of the olefinic carbons (C-3/3’) next to the quaternary carbons C-2/2’ (Figure 3).

Finally, the ^2^*J*_CH_ and ^3^*J*_CH_ HMBC cross-peaks from H-3/3’ (δ_H_ 4.98) to C-2/2’ (δ_C_ 78.2) and C-7/7’ (δ_C_ 44.8) together with HMBC from H_3_-9/9’ to C-4/4’ and from H_3_-10/10’ to C-5/5’ as well as ^4^*J*_CH_ from H_3_-10/10’ to C-6/6’ (δ_C_ 166.6) supported this assignment. The HMBC cross-peaks from H-3/3’ to the signals at 158.5 (C-4/4’) and 133.2 (C-5/5’) signify the ortho-placement of the OCH_3_ groups at C-4/4’ and C-5/5’ (Figure 3 and Figure 4). 

**Figure 5:**
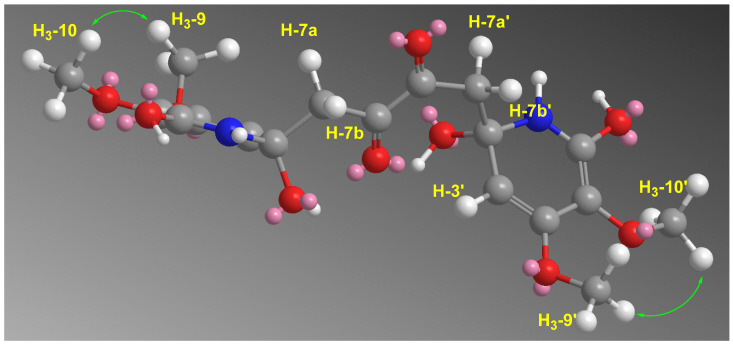
MM2-Energy minimized drawing of **1** displaying strong NOESY correlations between H_3_-9/9’ and H_3_-10/10’.

Finally, the ortho-substitutions of the methoxyl groups at C-4/4’ and C-5/5’ were confirmed from a NOESY experiment. The strong NOESY correlations observed between the methoxyl groups (H_3_-9/9’ and H_3_-10/10’) support their ortho-position at C-4/4’ and C-5/5’, respectively (Figure 5). Furthermore, the MM2-energy-minimized drawing of **1** (Figure 5) displayed strong correlations between H_3_-9/9’ and H_3_-10/10’ (Figure 5) confirming the ortho-substitutions of the methoxyl groups at C-4/4’ and C-5/5’. Thus, the 4,4’,5,5’-tetramethoxy substitutions in **1** were confirmed.

**Figure 6:**
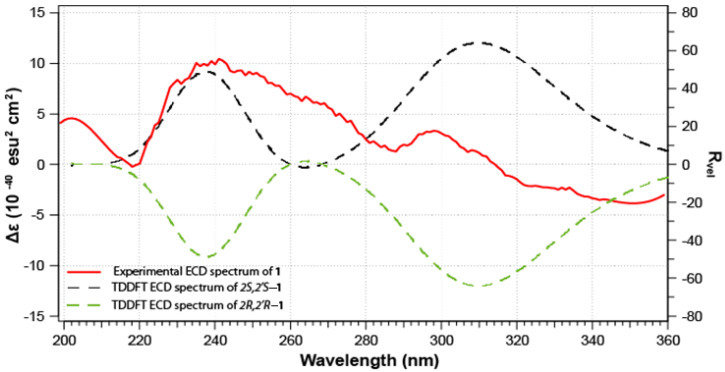
ECD spectra of fusaripyridine A (**1**).

The absolute configurations of the stereogenic carbons C-2 and C-2’ in **1** are established as 2*S* and 2’*S* as supported by comparison of the predicted and experimental ECD spectra (Figure 6). The theoretical ECD spectra (Figure 6) of compound **1** were calculated for a pair of configurations, 2*S*/2’*S* and 2*R*/2’*R,* as other combinations would result in a *meso* form. The computed configurations of 2S/2’S were well in agreement with the experimental spectrum; both positive Cotton Effect (CE) are observed in the predicted spectrum at ca. 240 and 310 nm. Thus, fusaripyridine A was assigned as 1,4-bis((*S*)-2,6-dihydroxy-4,5-dimethoxy-1,2-dihydropyridin-2-yl)butane-2,3-dione. 

### 2.3. Structure of Fusaripyridine B (**2**)

Fusaripyridine B (**2**) (Figure 2), an optically active compound, possesses a molecular formula C_18_H_24_N_2_O_12_ as supported by HRESIMS (Figure S8), being 32 mass unit large than **1**, suggesting additional two oxygen atoms in the molecule. Its ^13^C and ^1^H NMR spectra (Table 1) showed very close resonances to those of **1**, suggesting similar structures. Further, analyses of its ^1^H (Figure S9), ^13^C (Figure S10), NMR spectra, COSY (Figure S11), HSQC (Figure S12), HMBC (Figure S13) and NOESY (Figure S14) experiments confirmed its planar structure. Therefore, the oxygen atoms accounted for two additional hydroxy groups located at *N*-1 and *N*-1’, the sole positions allowed for substitution in **2**. 

Fusaripyridine B (**2**) shares the sign and magnitude of the optical rotation ([α]_D_ = +23°) with fusaripyridine A (**1**) ([α]_D_ = +27°). Therefore, we anticipate that **2** shares the biosynthetic pathway as **1** and possesses the same absolute configuration of 2S/2’S. Thus, fusaripyridine B was assigned as 1,4-bis((*S*)-1,2,6-trihydroxy-4,5-dimethoxy-1,2-dihydropyridin-2-yl)butane-2,3-dione. 

Natural *N*-substituted alkaloids are common secondary metabolites that reported from different terrestrial, marine and microbial organisms [25,26,27,28,29,30,31]. Reported substituents on the *N* atoms include OH, hydroperoxy, alkyl, heteroaromatic functionalities, and others [25,26,27,28,29,30,31]. Thus, fusaripyridine B represents an additional example of these alkaloids with *N,N’*-OH functionalities. 

### 2.4. Biological Evaluation of the Compounds

The fusaripyridines A (**1**) and B (**2**) are evaluated, in a disc diffusion assay, for their antimicrobial effects against *C. albicans*, *E. coli* and *S. aureus* at a concentration of 50 µg/disc. The compounds displayed potent antifungal effects against *C. albicans* with inhibition zones of 26 and 24 mm, and with MIC values of 8.0 and 8.0 µM, respectively. Further, the compounds displayed weak growth inhibition towards *S. aureus* and *E. coli* with MIC of ≥32.0 µM (Table 2). Moreover, fusaripyridines A (**1**) and B (**2**) showed weak effects towards HeLa cells with IC_50_ of ≥25 μM. The above data suggest the selectivity of the *C. albicans* towards fusaripyridines A and B.

## 3. Materials and Methods

### 3.1. General Experimental Procedures 

Optical rotations values were measured on a digital DIP-370 polarimeter (JASCO, Oklahoma City, OK, USA), while the UV spectra are obtained on a Hitachi 300 spectrometer (Hitachi High-Technologies Corporation, Kyoto, Japan). The ECD spectra were obtained on a JASCO J-810 spectropolarimeter (JASCO, Oklahoma City, OK, USA) with a 0.5 cm cell in MeOH. The NMR spectra were acquired on Bruker Avance DRX 600 MHz spectrometer (Bruker, Rheinstetten, Germany). Positive ion HRESIMS spectra were obtained on a Thermo LTQ Orbitrap XL mass spectrometer (Thermo Finnigan, Bremen, Germany). Normal SiO_2_ (Merck, Darmstadt, Germany) and Sephadex LH 20 (Pharmacia, Uppsala, Sweden) were used for chromatography. 

### 3.2. Host Organism, Suberea mollis

The Red Sea sponge *Suberea mollis* (Figure 1) was harvested by hand using scuba from Yanbu at the Saudi Red Sea at a depth of −30 m. Description of the sponge was reported earlier in details [32].

### 3.3. Preparation of the Fungal Isolate LY019

The fungal isolate *Fusarium* sp. LY019 was isolated from the Red Sea sponge *Suberea mollis* by culturing on Czapek-Dox yeast agar medium (K_2_HPO_4_ 0.1 g, MgSO_4_·7H_2_O 0.5 g, NaNO_3_ 3.0 g, KCl 0.5 g, FeSO_4_ 0.01 g, sucrose 30.0 g, agar 20.0 g, pH 6.7), using several purification steps until a pure isolate was obtained. After that, the isolate was inoculated into 50 mL of Czapek-Dox broth in 200 mL flasks at pH 7 and was incubated at 160 rpm, 25 °C for 7 days.

### 3.4. Preparation of Genomic DNA of Isolate LY019

Subculture of the isolate in corresponding broth for 4 days at 25 °C was carried out. Afterwards the mycelia were separated using a vacuum filtration unit. The mycelial mat was dried and powdered. The fungal DNA was obtained using QIAamp DNA Mini Kit (Qiagen, Hilden, Duesseldorf, Germany) as stated by the manufacturer.

### 3.5. Amplification of ITS-rDNA Fragments of Isolate LY019

Using ITS1 and ITS4 primers, the genomic DNA was served as the template for amplification of the fungal ITS-rDNA fragments [33]. The PCR reaction mixture for amplification and the reaction conditions are similar with previous work [22]. The agarose Gel DNA purification kit (Qiagen, Hilden, Duesseldorf, Germany) was used for purification of the PCR products.

### 3.6. Sequence of ITS-rDNA Regions of Isolate LY019

The sequence of the ITS-rDNA regions was compared with correlated sequences in NCBI [34]. Editing and alignment of the ITS-rDNA sequence with the best n-BLAST hits in GenBank were obtained using the Clustal X (version 1.83) program (Conway Institute, University College Dublin) [35]. The adjustment was achieved using BioEdit software (Bioz, Inc., Los Altos, Ca 94022, USA) manually [36]. MEGA 5 program was used for the base composition of the sequence [37]. 

### 3.7. Characterization of the Fungal Isolate LY019

The resulted sequence analysis exhibited 99% identity with *Fusarium commune* (NCIB accession number KU891512). 

### 3.8. Fermentation and Extraction of the Broth 

The fermentation was obtained in a 2.8 L Fernbach flasks with one liter of Czapek-Dox medium (Difco) containing 3% NaCl (*w*/*v*). Each flask was inoculated with 10 mL of the seed followed by incubation at 27 °C and 180 rpm for 10 days. After that, each flask was mixed with ethyl acetate (500 mL × 2) and shaken at 1800 rpm for 30 min. The organic phase was separated and dried under vacuum.

### 3.9. Purification of Fusaripyridines A and B

The dried extract (1.8 g) was partitioned on silica gel using hexane-CHCl_3_-MeOH gradients to give five fractions. The antifungal fraction (inhibition zone of 12 mm at 50 µg/disc against *C. albicans*) eluted with CHCl_3_-MeOH (98:2) (Fr. 5, 113 mg) was fractionated again on Sephadex LH-20 eluted with MeOH to give four major subfractions. Subfraction 4 (37.0 mg) was purified on a Gemini HPLC Column (5 µm C18 110 Å, 250 × 4.6 mm) using CH_3_CN-H_2_O (1:1) at 1.0 mL/min to give compounds **1** (t*_R_* = 4.3 min, 3.5 mg) and **2** (t*_R_* = 5.4 min, 1.4 mg).

#### Spectral Data of Fusaripyridines A and B

Fusaripyridine A (**1**): colorless solid; [α]_D_ = +27° (*c* 0.1, MeOH); UV (MeOH): λ_max_ (log ε): 252 (2.76) nm; NMR data: Table 1; HRESIMS: *m*/*z* 451.1332 (calcd for C_18_H_24_N_2_O_10_Na, [M + Na]^+^, 451.1323). 

Fusaripyridine B (**2**): colorless solid; [α]_D_ = +23° (*c* 0.1, MeOH); UV (MeOH): λ_max_ (log ε): 252 (2.76) nm; NMR data: Table 1; HRESIMS: *m*/*z* 483.1230 (calcd for C_18_H_24_N_2_O_12_Na, [M + Na]^+^, 483.1221). 

### 3.10. Computational Details

Calculations of the DFT are performed using Gaussian 16 W [38]. A conformation analysis using the GMMX plugin followed by a geometry optimization at the B3LYP/6-31g(d) level was conducted. Similarly, a frequency check at the same level of theory was performed. In addition, a 0.5 kcal.mol^−1^ cutoff was used to select only the most stable conformer. Rotational strengths were calculated on 20 excited states using the b3lyp/6-31g(d) level of theory. Finally, Gaussview 6 was used to plot the ECD spectra. The XYZ coordinates of the most stable conformer was added in the supporting data (Supporting data s2).

### 3.11. Antimicrobial Activities of the Compounds

The antimicrobial effects of **1** and **2** against *C. albicans*, *E. coli* and *S. aureus* were carried out at 50 µg/disc as previously reported in a disk diffusion assay [39,40,41,42].

### 3.12. Evaluation of the MIC 

Determination of the MIC of **1** and **2** against *C. albicans*, *E. coli* and *S. aureus* was performed in a macrodilution assay as described earlier [40,43].

### 3.13. MTT Assay

The antiproliferative and growth inhibition activities of **1** and **2** were carried out on HeLa cells (ATCC CCL-2) in MTT assay as reported earlier [44,45,46,47].

## 4. Conclusions

The organic extract of the fungus *Fusarium* sp. LY019 yielded two alkaloids, fusaripyridines A (**1**) and B (**2**), with an unprecedented 1,4-bis(2-hydroxy-1,2-dihydropyridin-2-yl)butane-2,3-dione backbone. Analyses of the spectroscopic data (NMR and HRESIMS) supported the planar structures of **1** and **2**, while the absolute configurations were obtained from the comparison of their ECD spectra. Fusaripyridines A and B are highly oxygenated alkaloids on the dihydropyridine moieties and represent the first candidates in this class. The compounds displayed selective and potent activities towards *C. albicans* with MIC values of 8.0 and 8.0 µM. The current results highlight the biosynthetic competences of fungi of marine origin as a foundation of drug leads with pharmaceutical potential. Fusaripyridines A and B could serve as a model for the discovery of novel antibiotics.

## Figures and Tables

**Table 1:** NMR data of fusaripyridines A (**1**) and B (**2**) (600 MHz for ^1^H and 150 for ^13^C, CD_3_OD).

Position	1				2			
	δ_C_, mult.	δ_H_ [m, *J* (Hz)]	HMBC	NOESY	δ_C_, mult.	δ_H_ [m, *J* (Hz)]	HMBC	NOESY
2, 2’	78.2, qC		H-3/3’, H_2_-7/7’		77.8, qC		H-3/3’, H_2_-7/7’	
3, 3’	99.9, CH	4.98 (s)	H_2_-7/7’		99.5, CH	4.97 (s)	H_2_-7/7’	
4, 4’	158.5, qC		H-3, H_2_-7, H_3_-9/9’		157.8, qC		H-3, H_2_-7, H_3_-9/9’	
5, 5’	133.2, qC		H-3/3’, H_3_-10/10’		132.9, qC		H-3/3’, H_3_-10/10’	
6, 6’	166.6, qC		H_2_-7/7’, H_3_-10/10’		166.6, qC		H_2_-7/7’, H_3_-10/10’	
7a, 7a’ 7b, 7b’	44.8, CH_2_	2.88 (d, 18.5) 2.19 (d, 18.5)	H-3/3’	H-7b/b’ H-7a/a’	44.5, CH_2_	2.88 (d, 18.5) 2.22 (d, 18.5)	H-3/3’	H-7b/b’ H-7a/a’
8, 8’	202.1, qC		H_2_-7/7’		202.0, qC		H_2_-7/7’	
9, 9’	59.6, CH_3_	4.01 (s)		H_3_-10/10’	59.5, CH_3_	3.99 (s)		H_3_-10/10’
10, 10’	52.4, CH_3_	3.82 (s)		H_3_-9/9’	52.4, CH_3_	3.81 (s)		H_3_-9/9’

**Table 2:** Antimicrobial activities of fusaripyridines A and B.

Compound	*C. albicans*		*E. coli*		*S. aureus*	
	Inhibition Zone(mm)	MIC (µM)	Inhibition Zone(mm)	MIC (µM)	Inhibition Zone(mm)	MIC (µM)
Fusaripyridine A (**1**)	26	8.0	9	≥32	9	≥32
Fusaripyridine B (**2**)	24	8.0	7	≥32	8	≥32
Ketoconazole ^a^	30	0.26	−	−	−	−
Ciprofloxacin ^b^	−	−	30	0.08	22	0.16

^a^ Positive antifungal drug; ^b^ positive antibacterial drug.

## Data Availability

Data is contained within the article or supplementary material.

## Supplementary Materials

The following are available online at https://www.mdpi.com/article/10.3390/md19090505/s1, Figures S1–S14: HRESIMS, ^1^HNMR, ^13^C NMR, DEPT, COSY, HSQC, HMBC, and NOESY spectra of fusaripyridines A (**1**) and B (**2**), XYZ coordinates of the most stable conformer of fusaripyridine A (**1**).
