# Supplementary material for: Fusaripyridines A and B; Highly Oxygenated Antimicrobial Alkaloid Dimers Featuring an Unprecedented 1,4-Bis(2-hydroxy-1,2-dihydropyridin-2-yl)butane-2,3-dione Core from the Marine Fungus Fusarium sp. LY019
Source: Mar Drugs. 2021 Sep 6;19(9):505. doi: 10.3390/md19090505 (PMC8471507; doi:10.3390/md19090505)

Supporting Information

Page

Figure S1. HRESIMS spectrum of fusaripyridine A (**1**) 2
Figure S2. 600 MHz ^{1}H NMR spectrum of fusaripyridine A (**1**) 3
Figure S3. 150 MHz ^{13}C NMR spectrum of fusaripyridine A (**1**) 4
Figure S4. ^{1}H-^{1}H COSY spectrum of fusaripyridine A (**1**) 5
Figure S5. Multiplicity-edited HSQC spectrum of fusaripyridine A (**1**) 6
Figure S6. ^{1}H-^{13}C HMBC spectrum of fusaripyridine A (**1**) 7
Figure S7. ^{1}H-^{1}H NOESY spectrum of fusaripyridine A (**1**) 8
Figure S8. HRESIMS spectrum of fusaripyridine B (**2**) 9
Figure S9. 600 MHz ^{1}H NMR spectrum of fusaripyridine B (**2**) 10
Figure S10. 150 MHz ^{13}C NMR spectrum of fusaripyridine B (**2**) 11
Figure S11. ^{1}H-^{1}H COSY spectrum of fusaripyridine B (**2**) 12
Figure S12. Multiplicity-edited HSQC spectrum of fusaripyridine B (**2**) 13
Figure S13. ^{1}H-^{13}C HMBC spectrum of fusaripyridine B (**2**) 14
Figure S14. ^{1}H-^{1}H NOESY spectrum of fusaripyridine B (**2**) 15

Figure S1. HRESIMS spectrum of fusaripyridine A (**1**).

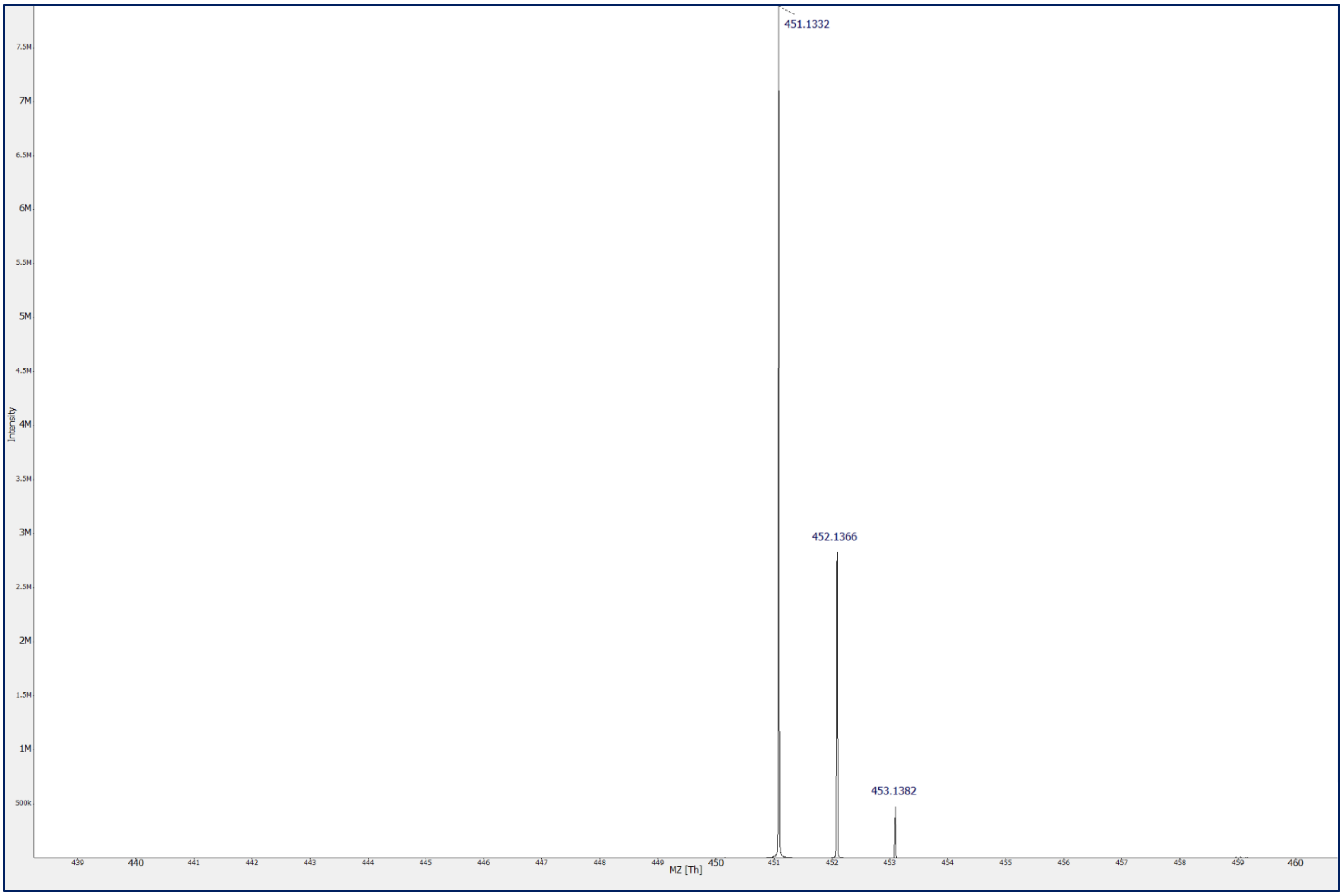

Figure S2. 600 MHz ^{1}H NMR spectrum of fusaripyridine A (**1**) (CD_3OD).

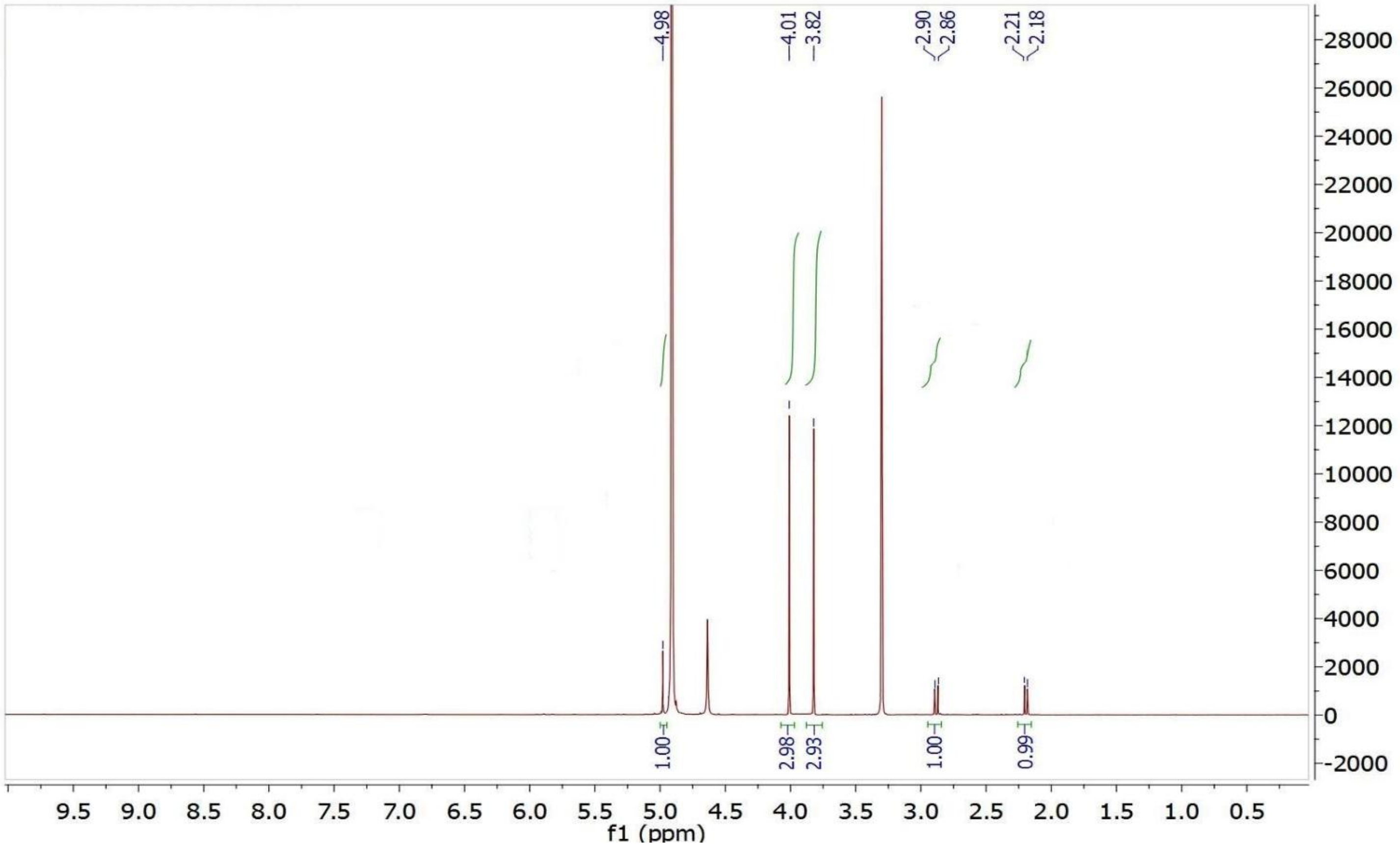

Figure S3. 150 MHz ^{13}C NMR spectrum of fusaripyridine A (**1**) (CD_3OD).

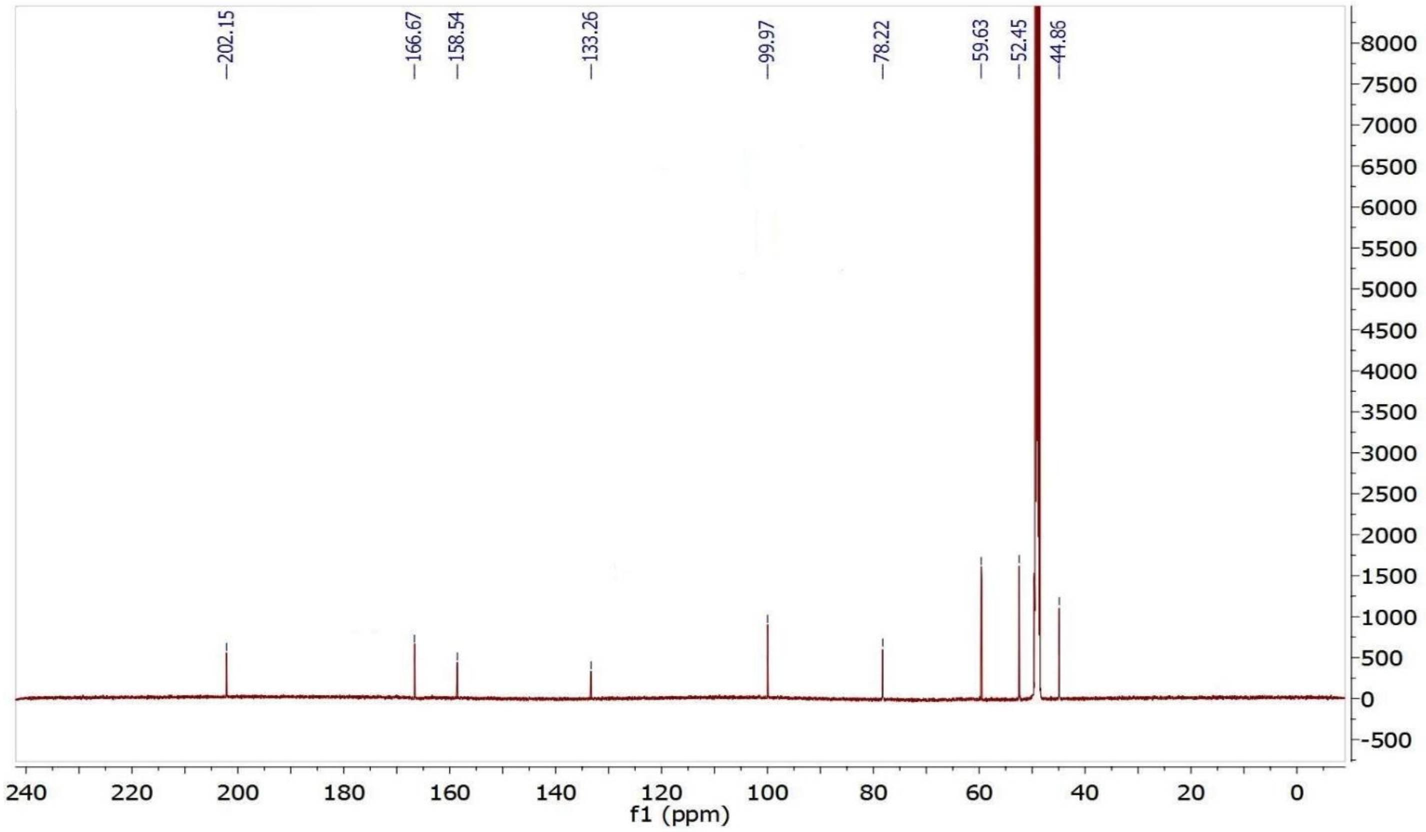

Figure S4. ^1H-^1H COSY spectrum of fusaripyridine A (**1**) (CD_3OD).

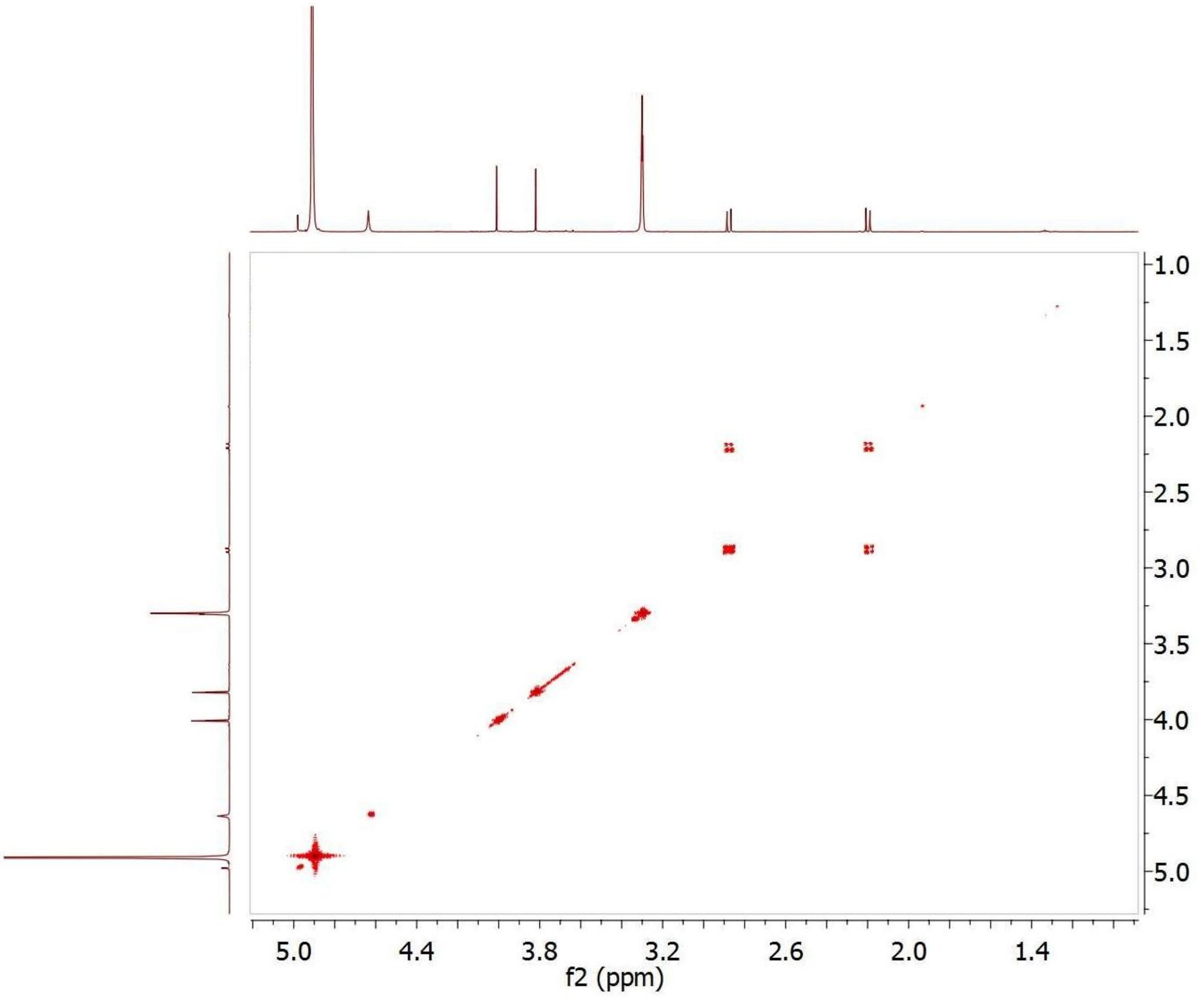

Figure S5. Multiplicity-edited HSQC spectrum of fusaripyridine A (**1**) (CD_3OD).

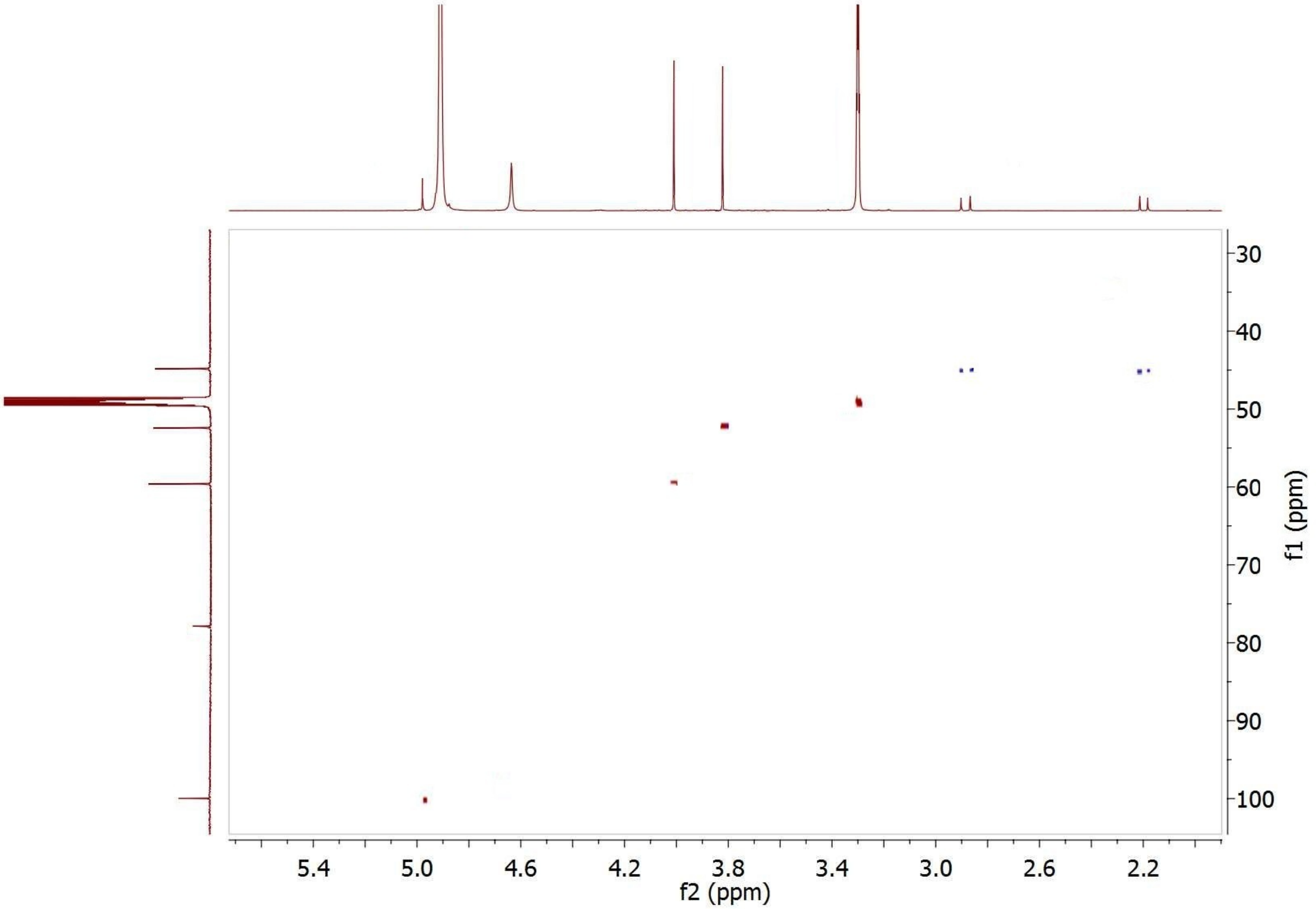

Figure S6. ^{1}H-^{13}C HMBC spectrum of fusaripyridine A (**1**) (CD_3OD).

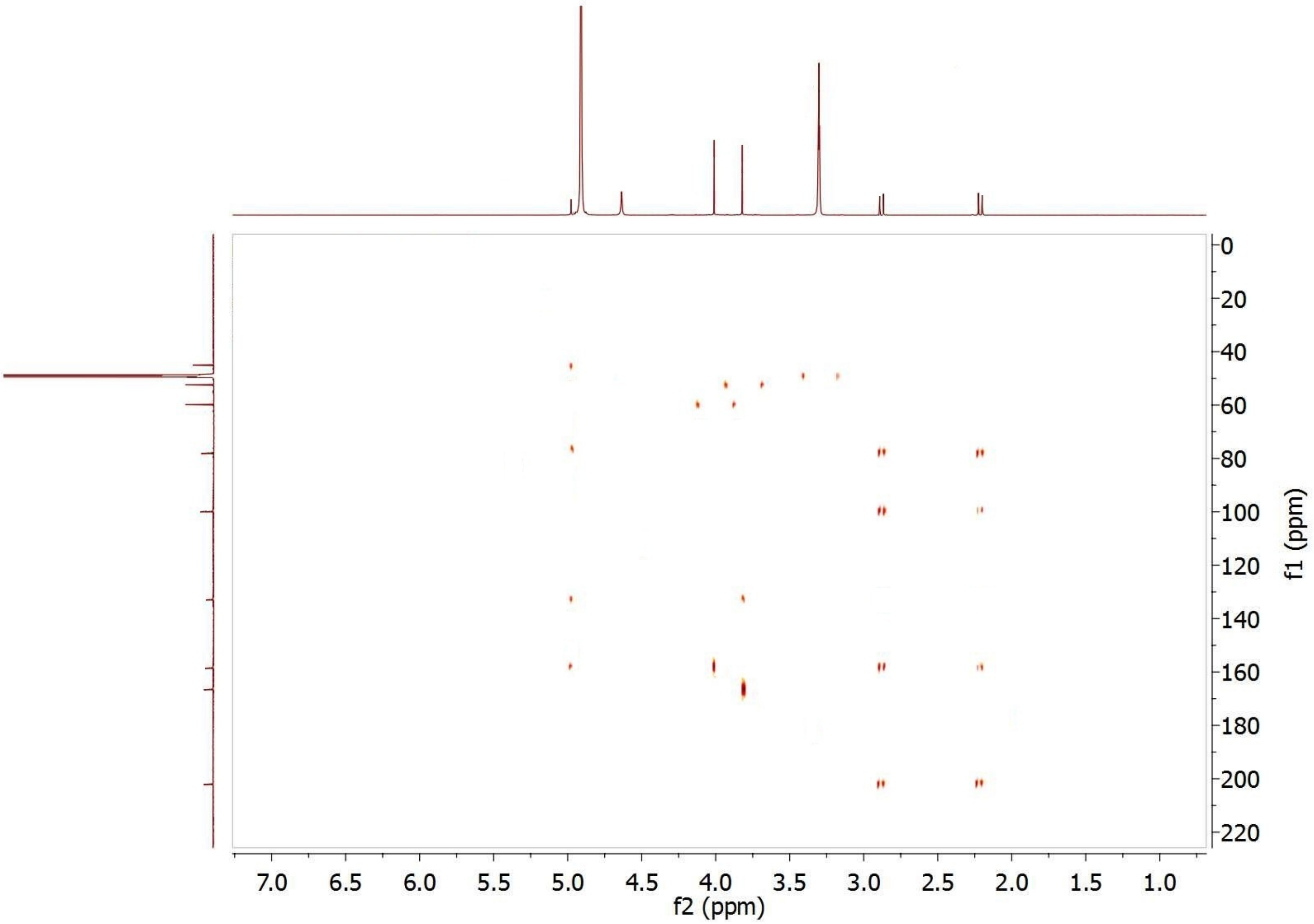

Figure S7. ^1H-^1H NOESY spectrum of fusaripyridine A (**1**) (CD_3OD).

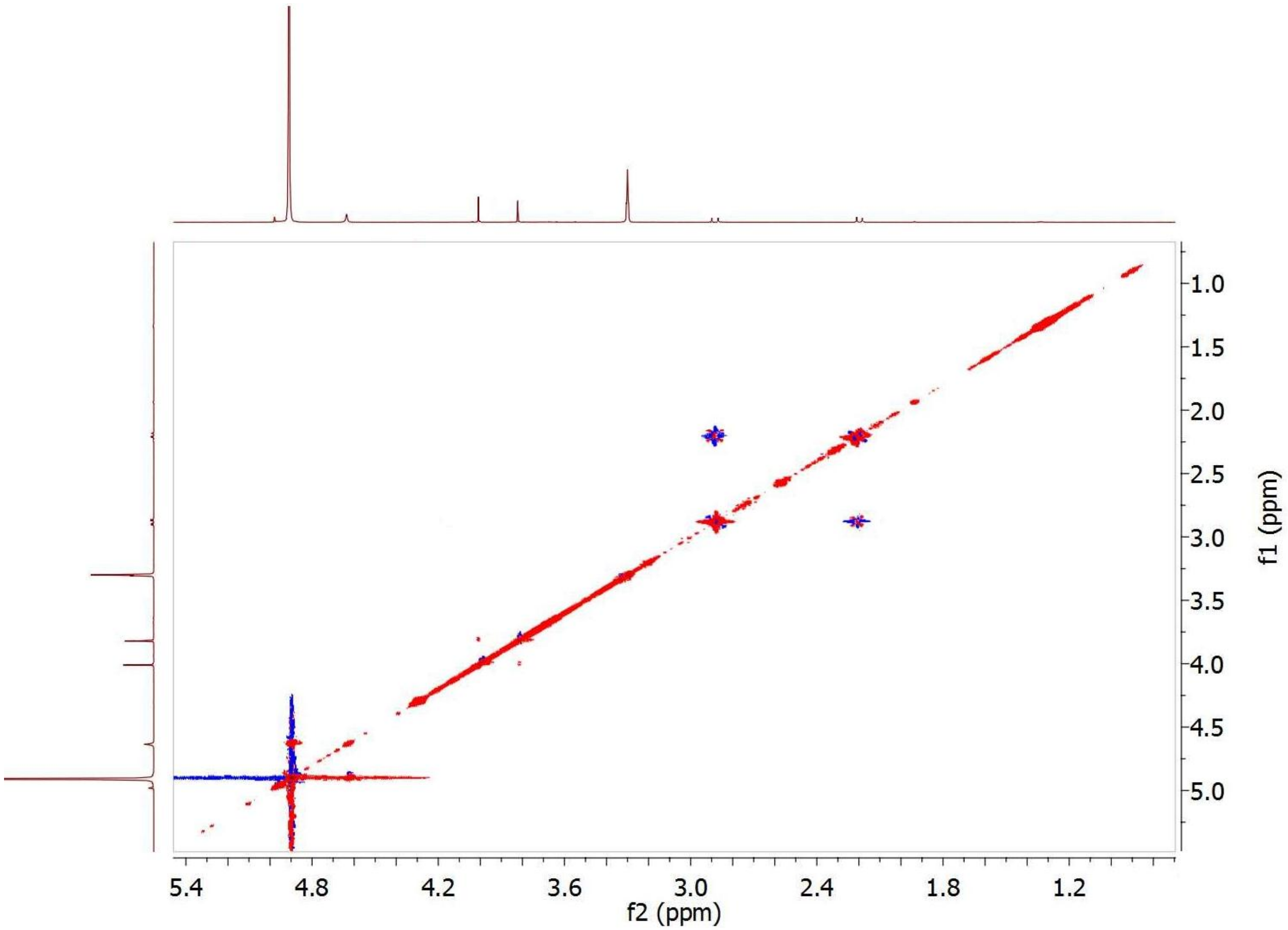

Figure S8. HRESIMS spectrum of fusaripyridine B (**2**).

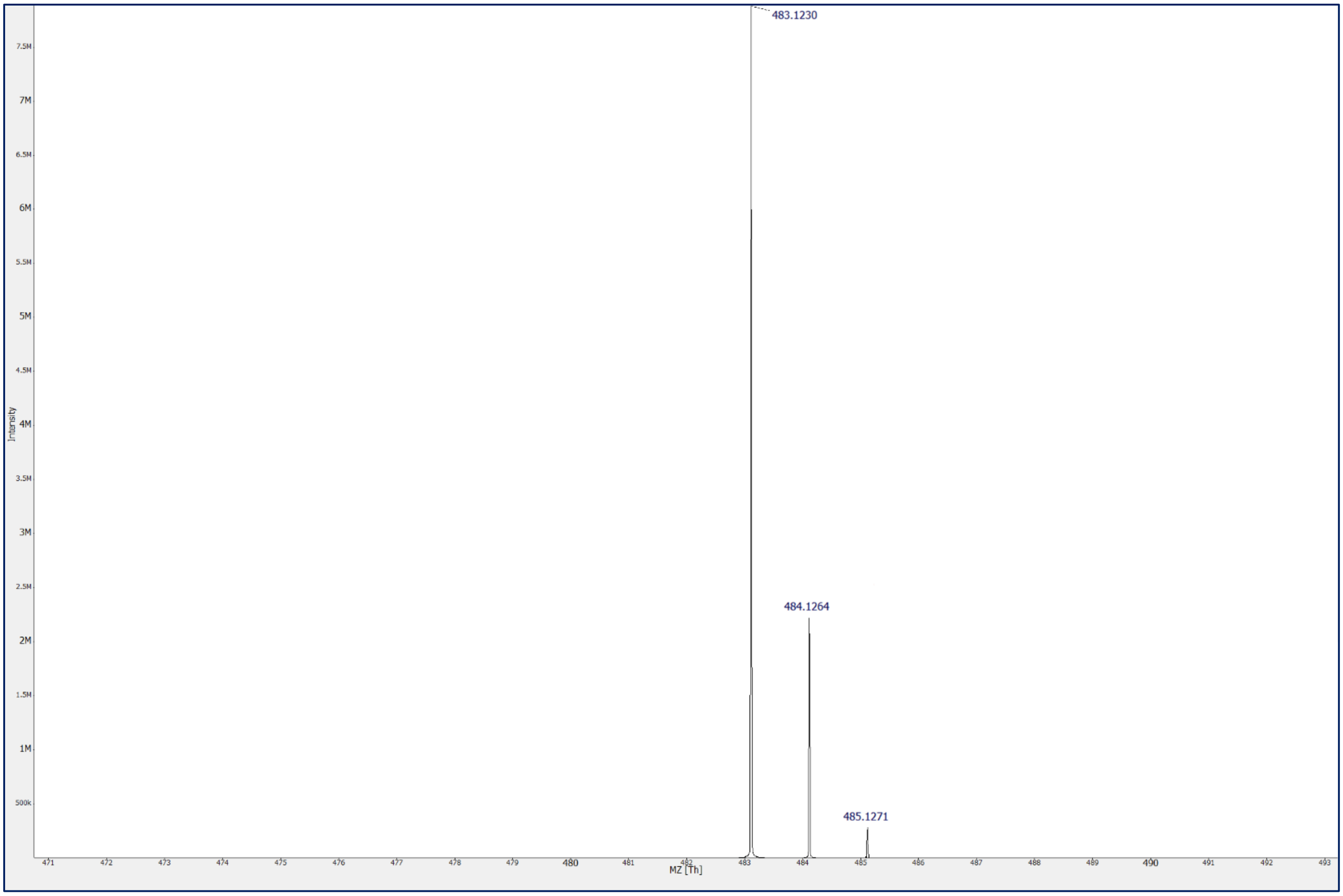

Figure S9. 600 MHz ^{1}H NMR spectrum of fusaripyridine B (**2**) (CD_3OD).

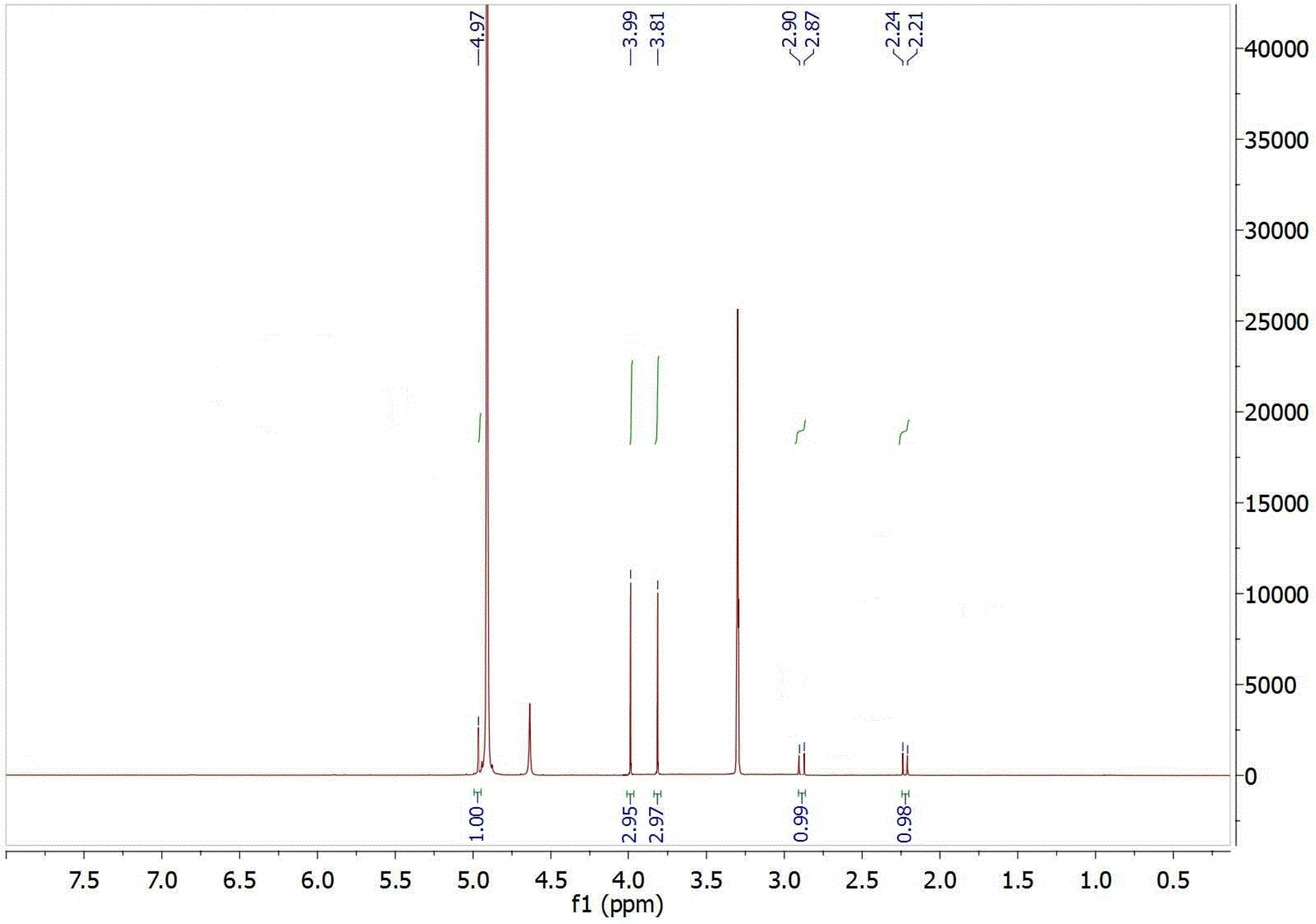

Figure S10. 150 MHz ^{13}C NMR spectrum of fusaripyridine B (**2**) (CD_3OD).

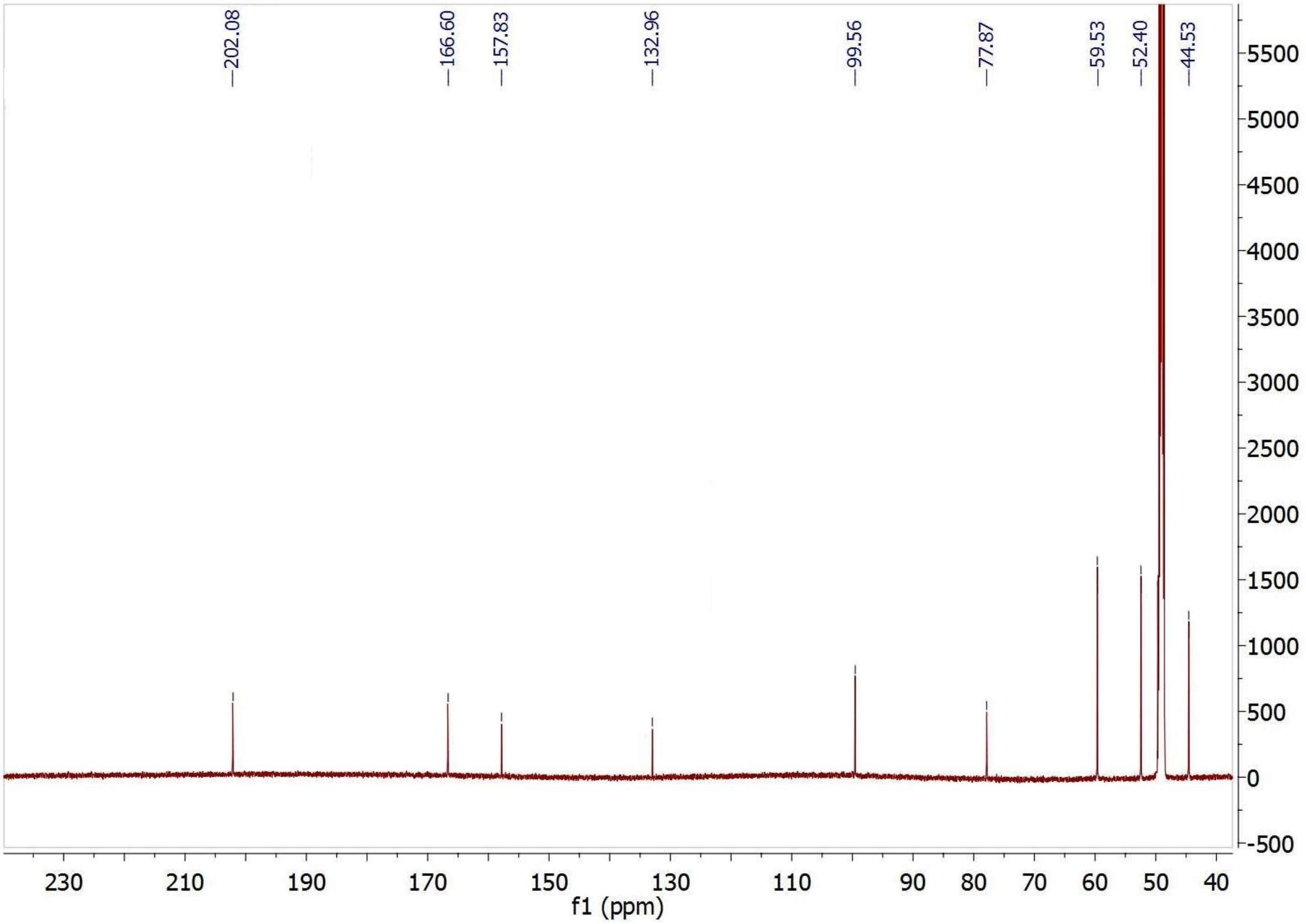

Figure S11. ^{1}H-^{1}H COSY spectrum of fusaripyridine B (**2**) (CD_3OD).

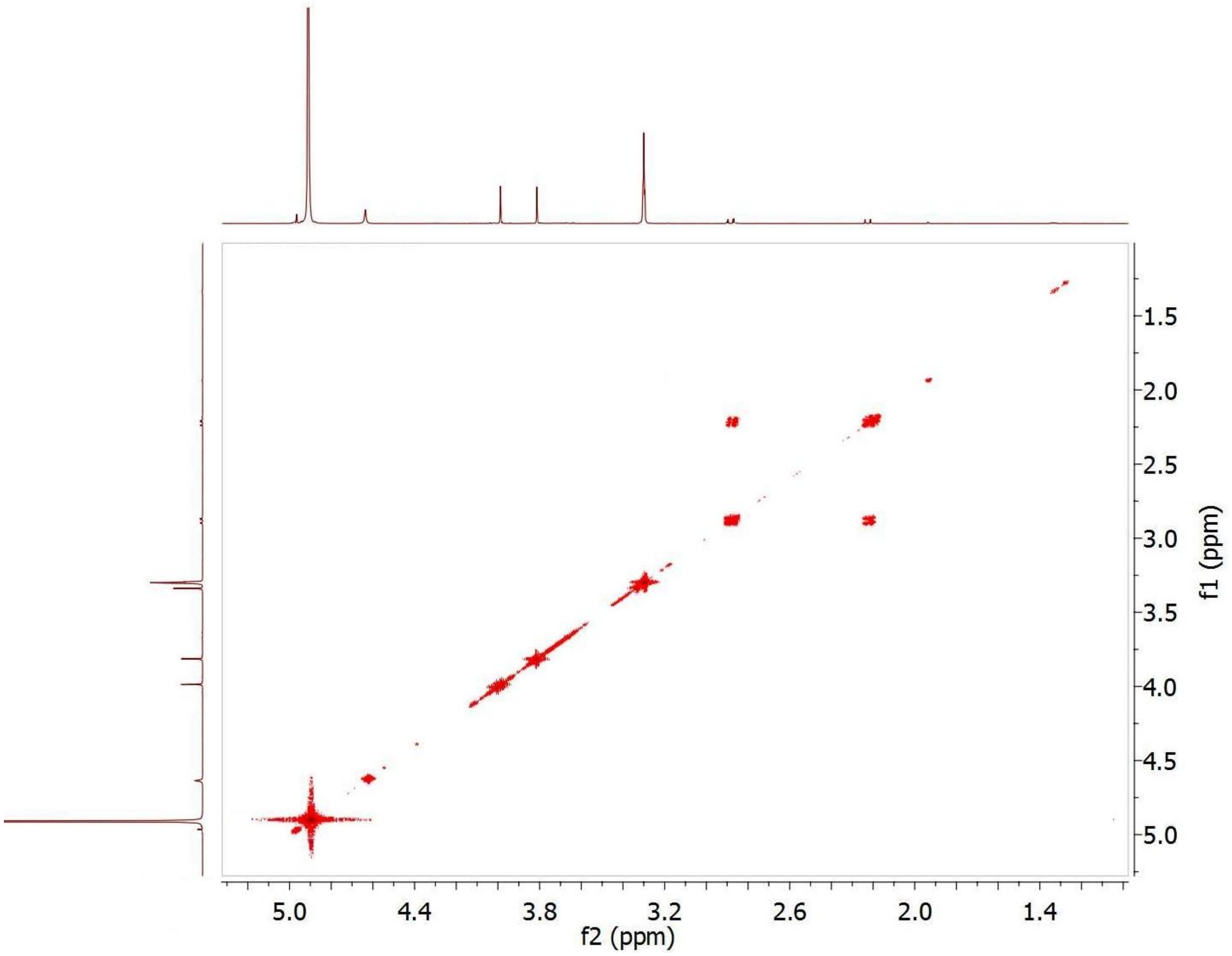

Figure S12. Multiplicity-edited HSQC spectrum of fusaripyridine B (**2**) (CD_3OD).

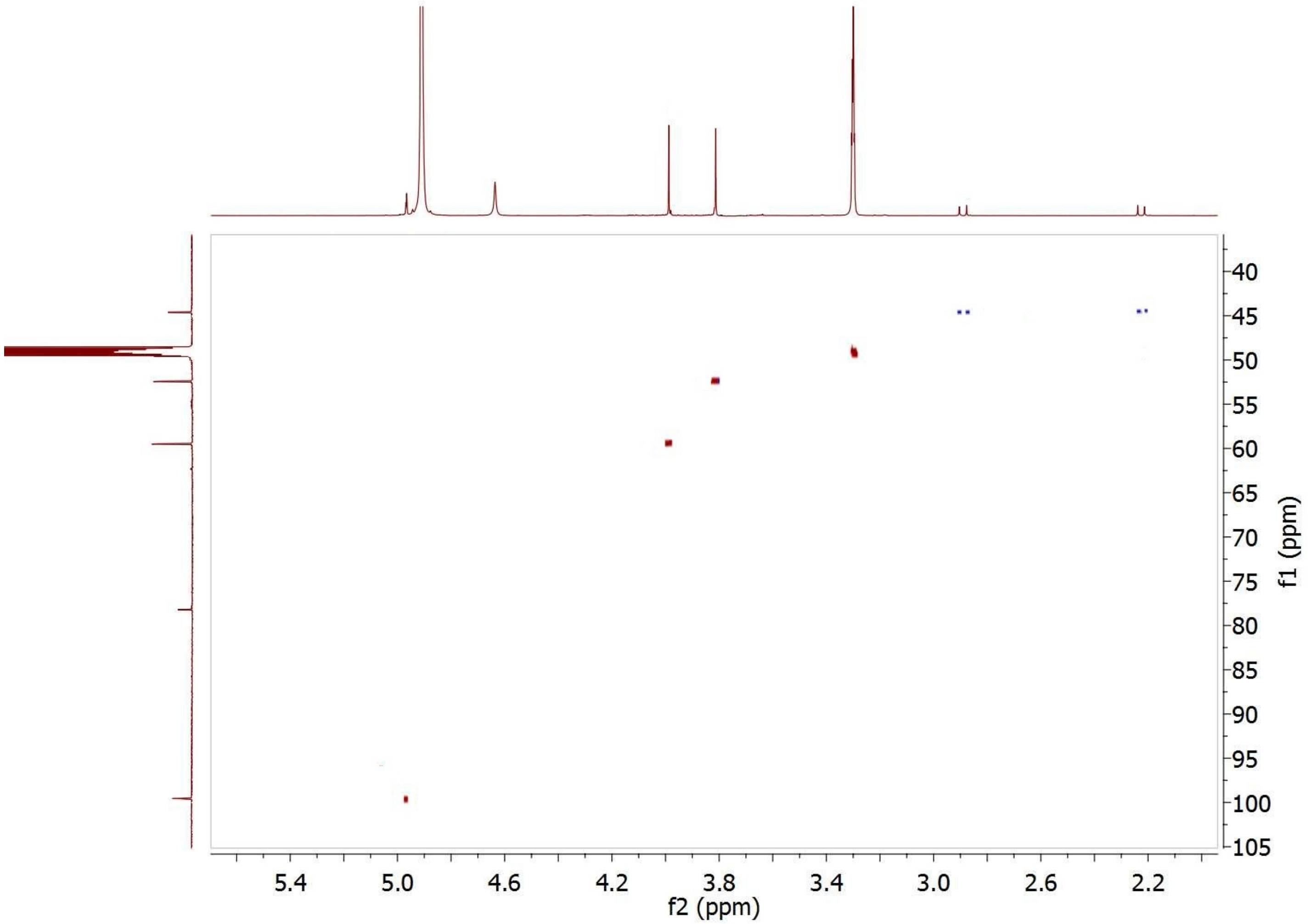

Figure S13. ^1H-^{13}C HMBC spectrum of fusaripyridine B (**2**) (CD_3OD).

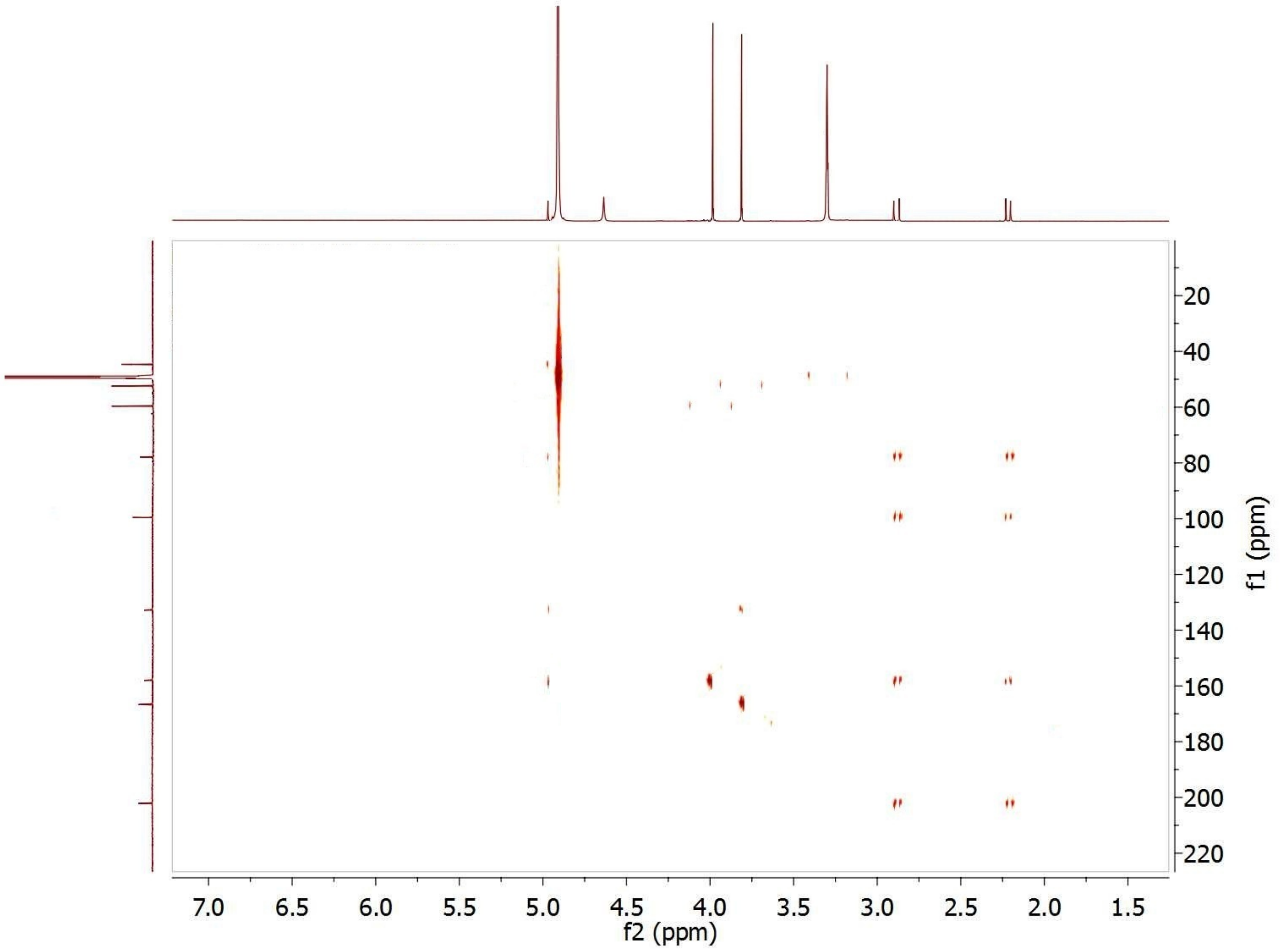

Figure S14. ^1H-^1H NOESY spectrum of fusaripyridine B (**2**) (CD_3OD).

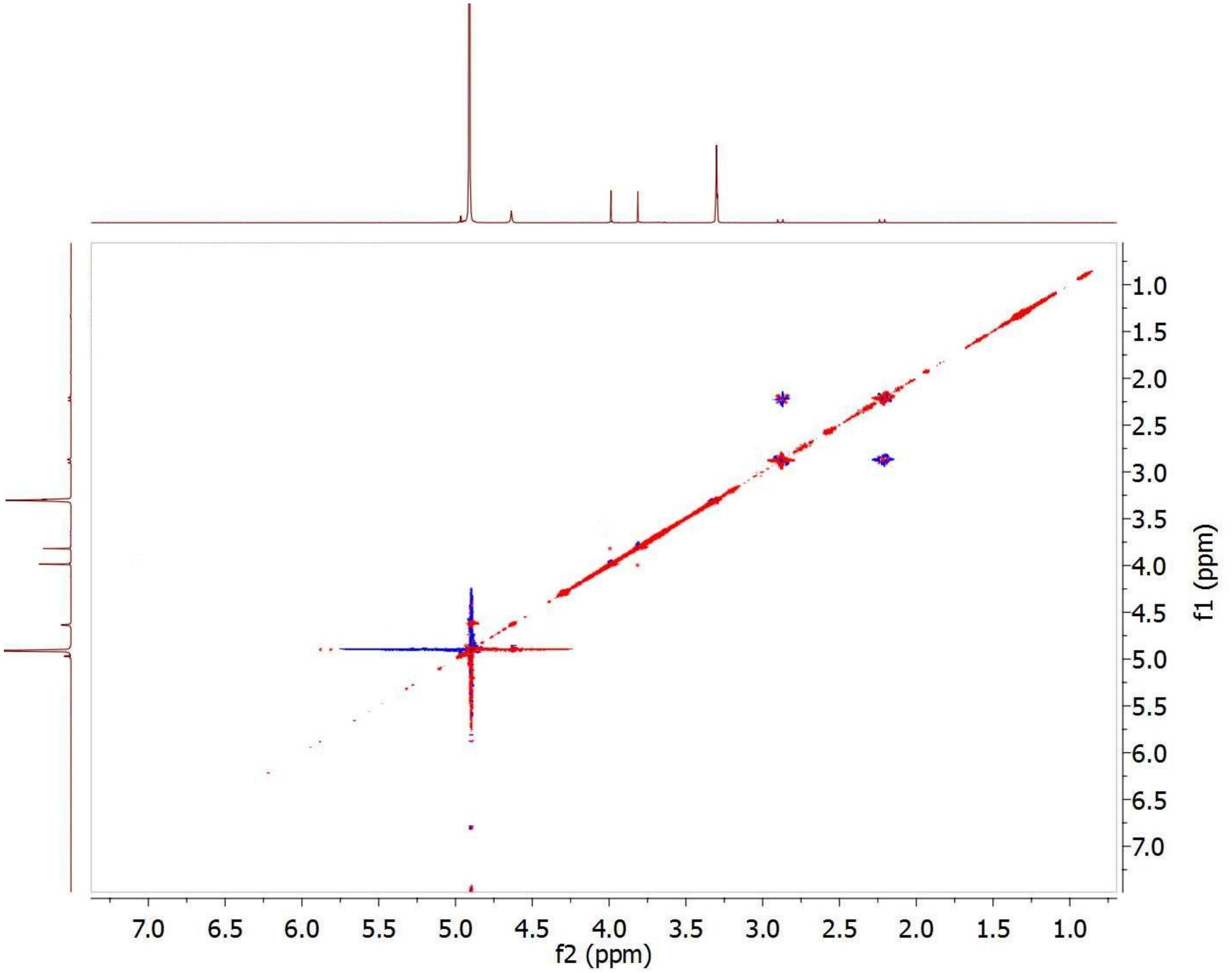

Supplement: Supplementary file 1 [file marinedrugs-19-00505-s001.zip › marinedrugs-1348122-SI/S1. Fusaripyridines_Supporting_Marine Drugs.pdf]
