# Supplementary material for: Fusaripyridines A and B; Highly Oxygenated Antimicrobial Alkaloid Dimers Featuring an Unprecedented 1,4-Bis(2-hydroxy-1,2-dihydropyridin-2-yl)butane-2,3-dione Core from the Marine Fungus Fusarium sp. LY019
Source: Mar Drugs. 2021 Sep 6;19(9):505. doi: 10.3390/md19090505 (PMC8471507; doi:10.3390/md19090505)
Supplement: Supplementary file 1 [file marinedrugs-19-00505-s001.zip › marinedrugs-1348122-SI/S2. XYZ coordinates of the most stable conformer of Fusaripyridine A (1).pdf]

Row	Highlight	Display	Tag	Symbol	X	Y	Z
1	No	Show	1	C	-4.4275540	-0.2896130	1.6774850
2	No	Show	2	O	-3.4886400	0.7560300	1.2313120
3	No	Show	3	C	-2.7318990	0.3565790	0.0883520
4	No	Show	4	C	-2.8608580	-0.8053520	-0.6084040
5	No	Show	5	C	-1.9125980	-1.2088560	-1.7642360
6	No	Show	6	O	-2.5139280	-1.0866120	-3.0818940
7	No	Show	7	C	-1.3615060	-2.6952970	-1.5030740
8	No	Show	8	C	-0.7983120	-2.8597160	-0.0511160
9	No	Show	9	O	-1.5125030	-2.9819440	0.9751680
10	No	Show	10	C	0.7956390	-2.8586710	0.0511600
11	No	Show	11	O	1.5101870	-2.9790110	-0.9751000
12	No	Show	12	C	1.3584770	-2.6950660	1.5033100
13	No	Show	13	C	1.9112960	-1.2093530	1.7648580
14	No	Show	14	O	2.5127090	-1.0882400	3.0826610
15	No	Show	15	C	2.8602980	-0.8069550	0.6092690
16	No	Show	16	C	2.7323440	0.3547120	-0.0881530
17	No	Show	17	O	3.4899110	0.7530960	-1.2309290
18	No	Show	18	C	4.4278630	-0.2936830	-1.6764650
19	No	Show	19	C	1.7539290	1.4067100	0.3514490
20	No	Show	20	O	1.5375050	2.6543950	-0.3734820
21	No	Show	21	C	2.8009040	3.3917750	-0.6354630
22	No	Show	22	C	0.8695740	1.1044900	1.3564890
23	No	Show	23	O	0.0226760	1.9286000	2.1071280
24	No	Show	24	N	0.6360110	-0.2998160	1.7485470
25	No	Show	25	O	0.1541570	-0.2552410	3.1564940
26	No	Show	26	N	-0.6360770	-0.3008900	-1.7479950
27	No	Show	27	O	-0.1546750	-0.2571130	-3.1561580
28	No	Show	28	C	-0.8685730	1.1039660	-1.3568080
29	No	Show	29	O	-0.0210300	1.9270160	-2.1078800
30	No	Show	30	C	-1.7526820	1.4075280	-0.3520110

31	No	Show	31	O	-1.5352440	2.6556110	0.3720270
32	No	Show	32	C	-2.7981920	3.3939760	0.6336560
33	No	Show	33	H	-3.9021550	-1.2395600	1.9073460
34	No	Show	34	H	-4.8950280	0.1051270	2.5991340
35	No	Show	35	H	-5.2182270	-0.4864190	0.9237110
36	No	Show	36	H	-3.6262080	-1.5432680	-0.3569660
37	No	Show	37	H	-1.6420210	-0.7502650	-3.5697370
38	No	Show	38	H	-2.1968870	-3.3986410	-1.6651740
39	No	Show	39	H	-0.5491900	-2.9095680	-2.2189180
40	No	Show	40	H	2.1931060	-3.3992760	1.6655390
41	No	Show	41	H	0.5457690	-2.9087030	2.2189170
42	No	Show	42	H	1.6414610	-0.7503510	3.5705080
43	No	Show	43	H	3.6253760	-1.5454360	0.3586650
44	No	Show	44	H	5.2183390	-0.4907960	-0.9225630
45	No	Show	45	H	3.9015660	-1.2432660	-1.9057850
46	No	Show	46	H	4.8957400	0.1001070	-2.5983150
47	No	Show	47	H	3.5375850	2.6975090	-1.0929710
48	No	Show	48	H	2.5444620	4.2175920	-1.3247140
49	No	Show	49	H	3.1931780	3.8022000	0.3160540
50	No	Show	50	H	-0.7215020	2.3371320	1.4194140
51	No	Show	51	H	-0.1349940	0.7784810	3.0823210
52	No	Show	52	H	0.1354420	0.7764130	-3.0824230
53	No	Show	53	H	0.7234040	2.3356400	-1.4204210
54	No	Show	54	H	-3.1902000	3.8041460	-0.3180760
55	No	Show	55	H	-2.5411650	4.2199870	1.3224520
56	No	Show	56	H	-3.5352820	2.7004150	1.0915730

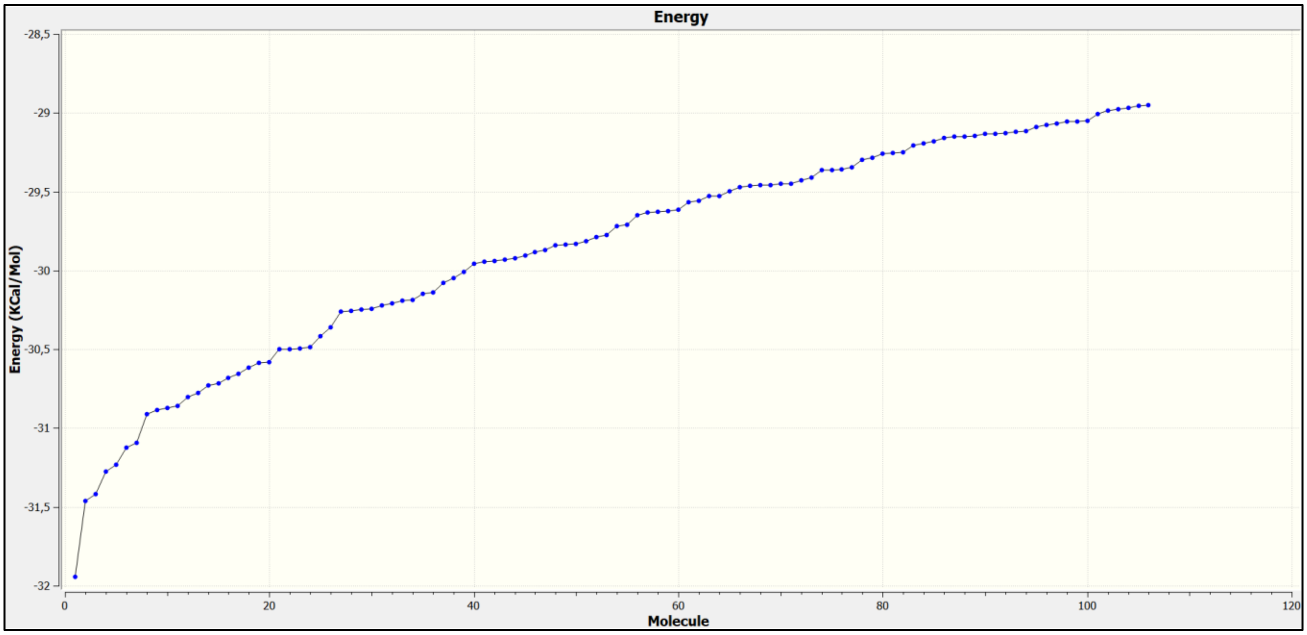

The GMMX plugin for the conformational analysis at a 0.5 kcal.mol^{-1} cutoff was used to select only the most stable conformer (cf image).
